# Statistics of Natural Binaural Sounds

**DOI:** 10.1371/journal.pone.0108968

**Published:** 2014-10-06

**Authors:** Wiktor Młynarski, Jürgen Jost

**Affiliations:** 1 Max-Planck Institute for Mathematics in the Sciences, Leipzig, Germany; 2 Santa Fe Institute, Santa Fe, New Mexico, United States of America; Harvard Medical School/Massachusetts General Hospital, United States of America

## Abstract

Binaural sound localization is usually considered a discrimination task, where interaural phase (IPD) and level (ILD) disparities at narrowly tuned frequency channels are utilized to identify a position of a sound source. In natural conditions however, binaural circuits are exposed to a stimulation by sound waves originating from multiple, often moving and overlapping sources. Therefore statistics of binaural cues depend on acoustic properties and the spatial configuration of the environment. Distribution of cues encountered naturally and their dependence on physical properties of an auditory scene have not been studied before. In the present work we analyzed statistics of naturally encountered binaural sounds. We performed binaural recordings of three auditory scenes with varying spatial configuration and analyzed empirical cue distributions from each scene. We have found that certain properties such as the spread of IPD distributions as well as an overall shape of ILD distributions do not vary strongly between different auditory scenes. Moreover, we found that ILD distributions vary much weaker across frequency channels and IPDs often attain much higher values, than can be predicted from head filtering properties. In order to understand the complexity of the binaural hearing task in the natural environment, sound waveforms were analyzed by performing Independent Component Analysis (ICA). Properties of learned basis functions indicate that in natural conditions soundwaves in each ear are predominantly generated by independent sources. This implies that the real-world sound localization must rely on mechanisms more complex than a mere cue extraction.

## Introduction

The idea that sensory systems reflect the statistical structure of stimuli encountered by organisms in their ecological niches [1]–[3] has driven numerous theoretical and experimental studies. Obtained results suggest that tuning properties of sensory neurons match regularities present in natural stimuli [4]. In light of this theory, neural representations, coding mechanisms and anatomical structures could be understood by studying characteristics of the sensory environment.

Natural stimuli have a very different and richer structure than standard sensory input used in experiments. They are typically noisier and generated by multiple interferring sources (a good auditory example is the famous “cocktail party problem” [5]). The statistical characterization of ecological input allows to better understand the complexity of perceptual tasks, when performed in non-laboratory conditions. This in turn provides a constraint on a class of algorithms, which may be implemented by the nervous system when dealing with the real world stimuli.

To date, natural scene statistics research have been focusing mostly on vision [6]. Nevertheless, a number of interesting results relating properties of natural sound to the auditory system have also been delivered. For instance, Rieke et al demonstrated that auditory neurons in the frog increase information transmission, when the spectrum of the white-noise stimulus is shaped to match the spectrum of a frog call [7]. In a more recent experiment, Hsu and colleagues [8] shown similar facilitation effects in the zebra finch auditory system using stimuli with power and phase modulation spectrum of a conspecific song. Modulation spectra of natural sounds were shown to display a characteristic statistical signature. This observation allowed to form quantitative predictions about neural representations and coding of sounds [9]. Other statistical models of natural auditory scenes have also led to interesting observations. Low-order, marginal statistics of amplitude envelopes, for instance, seem to be preserved across frequency channels as shown by Attias and Schreiner [10]. This means that all locations along the cochlea may be exposed to (on average) similar stimulation patterns in the natural environment. A strong evidence of adaptation of the early auditory system to natural sounds was provided by two complementary studies by Lewicki [11] and Smith and Lewicki [12]. The authors modeled high order statistics of natural stimuli by learning sparse representations of short sound chunks. In such way, they reproduced filter shapes of the cat's cochlear nerve. These results were recently extended by Carlson et al [13] who obtained features resembling spectro-temporal receptive fields in the cat's Inferior Colliculus by learning sparse codes of speech spectrograms. This constitutes a strong suggestion that neural representations of acoustic stimuli reflect structures present in the natural environment.

The above mentioned studies investigated statistical properties of single channel, monaural sounds relating them to the functioning of the nervous system. However, in natural hearing conditions the sensory input is determined by many additional factors - not only properties of the sound source. Air pressure waveforms reaching the cochlea are affected by positions and motion patterns of sound sources as well as head movements of the listening subject. These spatial aspects of the environment generate differences between stimuli present in each ear (i.e. interaural differences). The wavefront of a sound reaches firstly the ear ipsilateral to its source, and after a short time interval the contralateral one. The resulting delay is known as the interaural time difference (ITD), and in narrowly tuned frequency channels it corresponds to the interaural phase difference (IPD). Additionally, sound received by the contralateral ear is attenuated by the head, which generates an interaural level difference (ILD). According to the widely acknowledged duplex theory [14], [15], in mammals, IPDs are used to localize low frequency sounds. The theory predicts that in higher frequency regimes IPDs become ambiguous and therefore sounds of frequency above a certain threshold (around 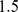 kHz in humans) are localized based on ILDs which become more pronounced due to the low-pass filtering properties of the head. Binaural hearing mechanisms have also been studied in terms of adaptation to stimulus statistics. Harper and McAlpine [16] have shown that tuning properties of IPD sensitive neurons in a number of species can be predicted from distributions of this cue naturally encountered by the organism. This was done by forming a model neuronal representation of maximal sensitivity to the stimulus change, as quantified by the Fisher information.

Existing evidence suggests that in order to increase the coding efficiency, the auditory system adapts to variation in binaural cue statistics. Therefore to understand its functioning in natural hearing conditions it is important to know, what are the typically encountered distributions of binaural cues. Understanding the distribution variability across different types of natural scenes is crucial as well, since it determines how flexible neuronal adaptation mechanisms should be. Binaural sound statistics determine also the complexity of the sound localization task. Natural sounds are typically generated by multiple independent sources, scattered in different configurations at both sides of the head. In such cases, binaural cues do not correspond to a position of a single object - its identification has to rely on algorithms more complicated than those useful in a simple, laboratory setting. One could assess to which extent this is the case in real auditory scenes, by quantifying the degree of dependence of sounds in each ear.

This paper attempts to answer the above-mentioned questions. Firstly it characterizes marginal statistics of binaural cues encountered in natural hearing conditions, which to our best knowdledge, has not been done previously. Secondly, it analyzes the redundance of monaural waveforms and in this way estimates the difficulty of a sound localization task in real environments. To achieve those goals we performed binaural recordings of three real-world auditory scenes of different acoustic and spatial characteristics. In the next step we extracted binaural cues - IPDs and ILDs and studied their marginal distributions. Using Independent Component Analysis (ICA), we show that in real-world auditory scenes, monaural waveforms are mutually much less interdependent than in a simple, point-source case. Present results provide a step towards understanding the mechanisms of spatial hearing in ecological conditions.

## Results

### Recorded scenes

**Figure 1:**
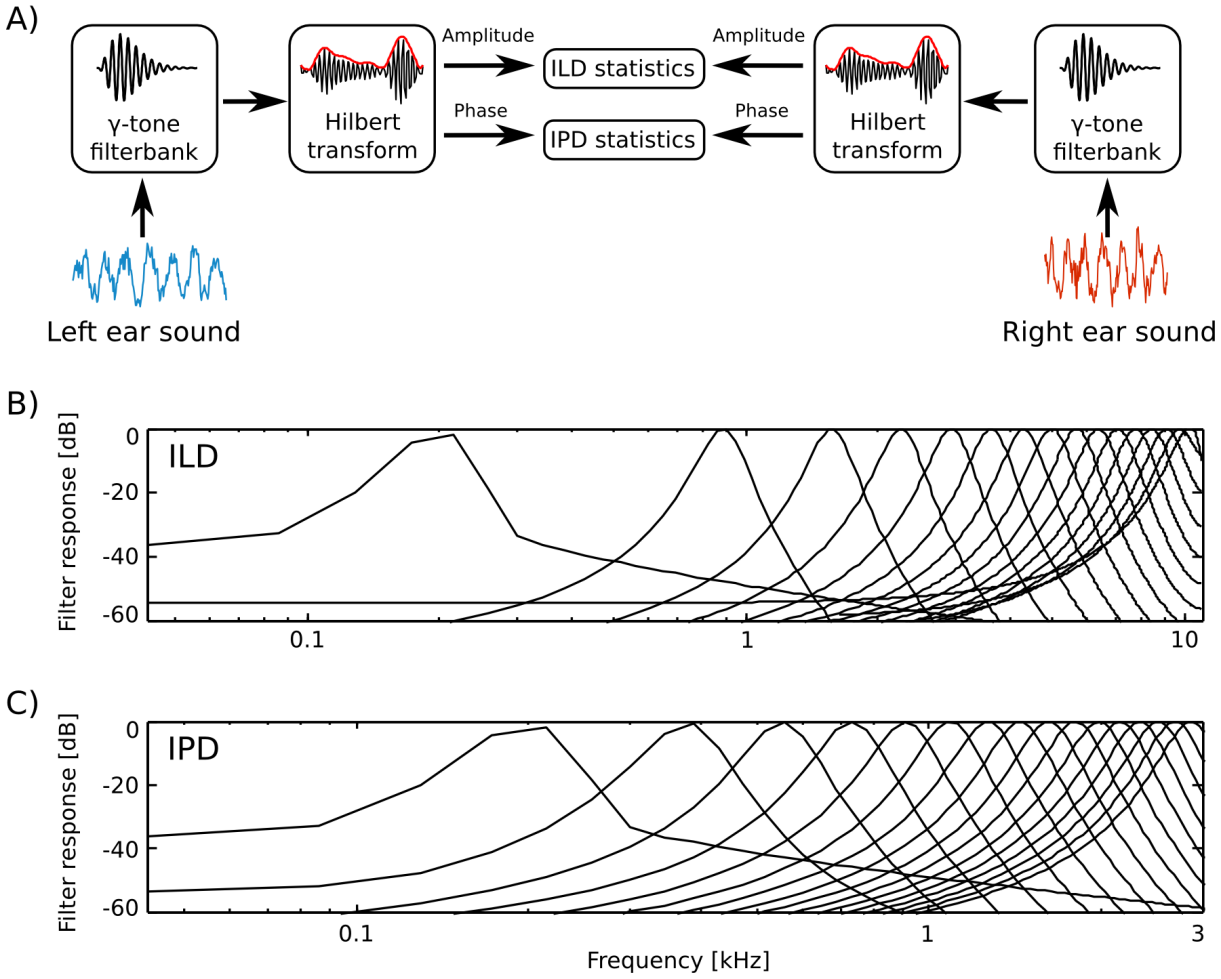
Preprocessing and cue extraction pipeline. A) Preprocessing scheme. Raw sounds in each ear were tranformed using a cochleotopic filterbank. In the next step the Hilbert transform was computed to separate amplitude from phase. Finally IPDs and ILDs were extracted. B) Filter response spectra of 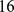 out of 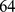 filters used to extract interaural level differences. C) Filter response spectra of 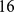 out of 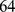 filters used to extract interaural phase differences.

In this work we analyzed 12 minute recordings of three different auditory scenes - nocturnal nature, forest walk and city center (the analysis pipeline is depicted on figure 1). The scenes were selected as representative examples of a broad range of possible acoustic environments. In each scene multiple sound sources positioned at a diverse set of locations were present. Sound types and spatial configuration of sources however, varied from scene to scene. In the nocturnal nature recording, the recording subject was static, and the scene was dominated by calls of grasshoppers (which do not move while generating sound). This recording was an example of a scene, where many non-moving sources are present, and their joint activity results in an ambient sound. The forest walk scene was much less stationary - the subject was freely moving in a wooded area while talking to another person. The scene included speech, ambient environmental sound sources (wind, leaves, stream) as well as transient ones (wood cracks, steps). This case was used as an example of a scene, where binaural information is affected by the motion and speech of the listening subject. In the third scene - city center - the subject was again listening passively, and the sensory input was rapidly changing due to the presence and the constant motion of multiple human speakers. This recording exemplified very dynamic auditory scenes with numerous moving sources.

Auditory environments chosen for recording were different from each other. We attempted to obtain representative samples of three classes of auditory scenes categorized by spatial configurations - static sources (nocturnal nature), moving sources (city center), and moving subject (forest walk). A statistical variation among examples analyzed here should therefore capture variability across numerous other cases.

Scene selection in this study did not include all possible cases. For instance no recording was performed in an enclosed, highly reverberant environment. Additionally all recordings were done in similar weather conditions, which may have narrowed the range of stimulus properties. The nocturnal nature and city center scenes consisted of constantly active sources - no periods of silence were present, which happens in natural hearing conditions. Despite those limitations, we argue that current data are heterogenous enough to draw general conclusions.

While auditory scenes were selected as a representation of diverse environments, recordings of each scene were performed in an unbiased way. Position of the subject was chosen at random in the two static recordings, and his motion was not constrained or pre-designed while walking. In this way, samples of a typical sensory input were collected. By refraining from recording in carefully controlled settings, where some feature (for instance loudness) in each ear would be the same, we avoided the selection bias. Natural auditory scenes are rarely spatially symmetric and stimuli analyzed here provide examples of what one typically hears. Understanding the structure of unbiased rather than fine-tuned stimuli should give better insights into the functioning of the nervous system in natural conditions [17].

### Sound spectra

**Figure 2:**
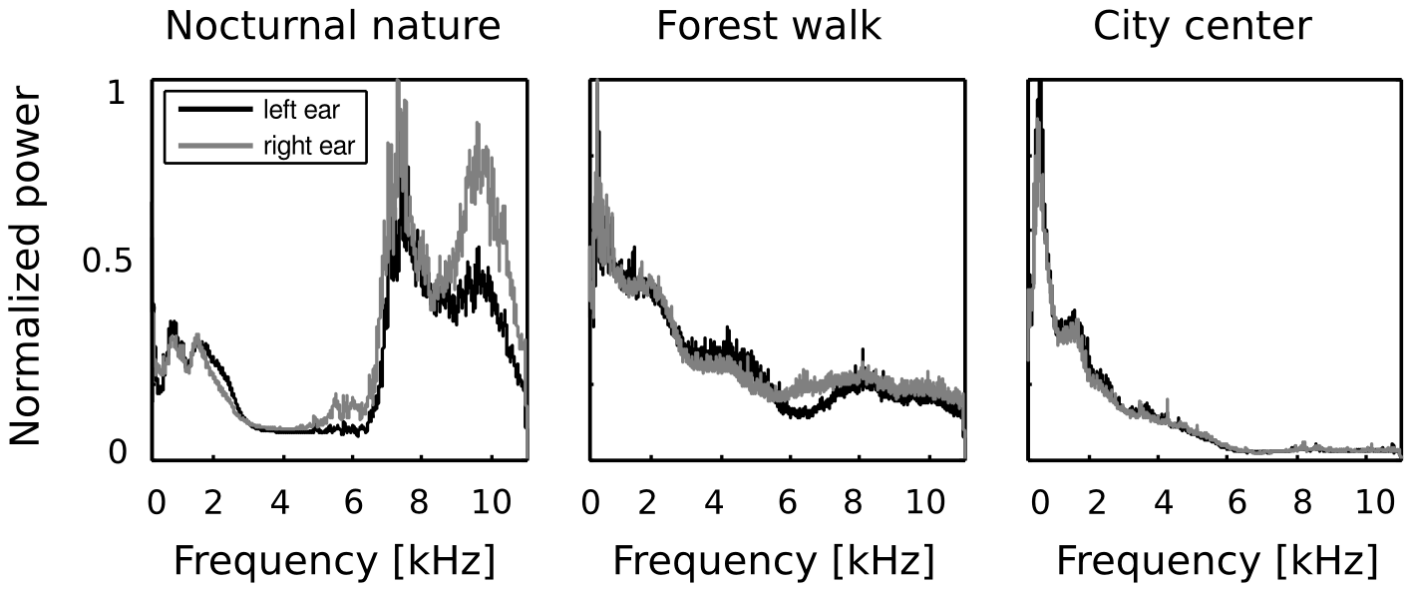
Frequency spectra of binaural recordings. In the forest walk and the city center scenes spectra of sounds in the left and in the right ear (black and gray lines respectively) were approximately the same. In the nocturnal nature scene, a sound source was constantly present on the right side of the head, therefore more power was present in high frequencies in the right ear.

Frequency spectra of recorded sounds are displayed on figure 2. Strong differences in spectrum across all recorded auditory scenes was present. In two of them - the forest walk scene and the city center scene, frequency spectrum had an exponential (power-law) shape, which is a characteristic signature of natural sounds [18]. Since the nocturnal nature scene was dominated by the grasshoper sounds, its spectrum had two dominant peaks around 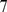 and 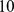 kHz. Sounds in both ears contained similar amount of energy in lower frequencies (below 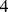 kHz) - which is reflected by a good overlap of monaural spectra on the plots. In higher frequencies though, the spectral power was not equally distributed in both ears. This difference is most strongly visible in the spectrum of the nocturnal nature scene. There, due to a persistent presence of a sound source (a grasshoper) closer to the right ear, corresponding frequencies were amplified with respect to the contralateral ear. Since the spatial configuration of the scene was static, this effect was not balanced by being averaged out in time. Monaural spectra of the forest walk scene overlapped to a much higher degree. A small notch in the left ear spectrum is visible around 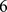 kHz. The city center scene, has almost identical monaural spectra. This is a reflection of its rapidly changing spatial configuration - sound sources of similar quality (mostly human speakers) were present in all positions during the time of the recording.

### Interaural level difference statistics

**Figure 3:**
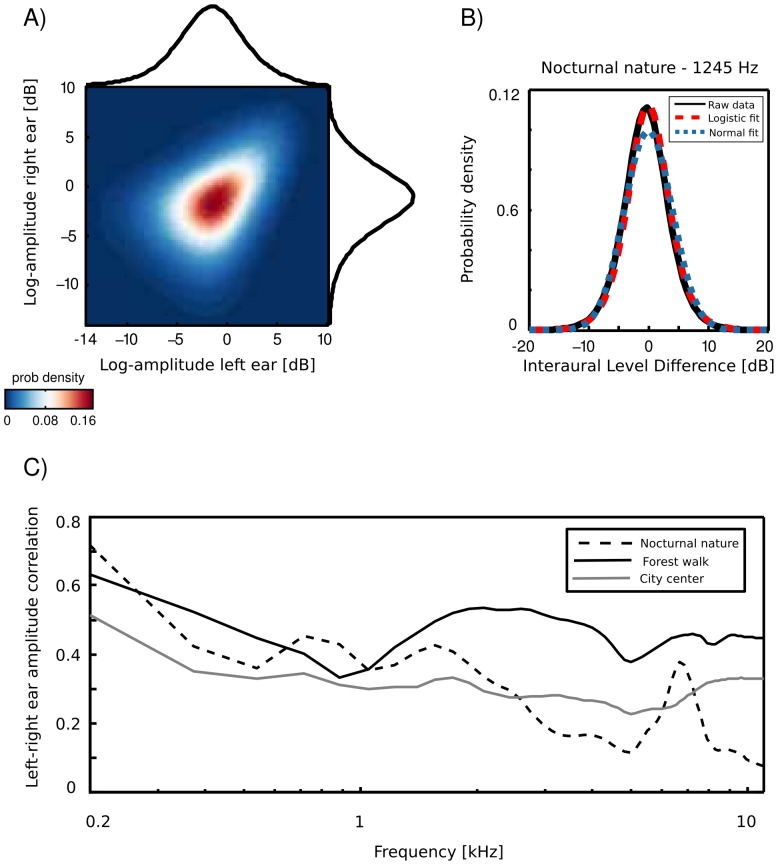
Binaural amplitude statistics. A) An exemplary joint distribution of monaural amplitudes at 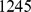 Hz. Exemplary data were taken from the nocturnal nature recording. B) An ILD distribution of the same data, plottedtoghether with a Gaussian and a logistic fit (blue and red dotted lines respectively) C) Interaural amplitude correlations across frequency channels

An example joint amplitude distribution in the left and the right ear is depicted on figure 3 A. It is not easily described by any parametric probability density function (pdf), however monaural amplitudes reveal a strong linear correlation. Correlation coefficient can be therefore used as a simple measure of interaural redundancy by indicating how similar the amplitude signal in both ears is, at a particular frequency channel. Interaural amplitude correlations for all recorded scenes are plotted as a function of frequency on figure 3 C. A general trend across the scenes is that correlations among low frequency channels (below 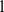 kHz) are strong (larger than 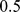) and decay with increasing frequency. Such trend is expected due to the filtering properties of the head, which attenuates low frequencies much less than higher ones. The spatial structure of the scene also finds reflection in binaural correlation - for instance, a peak is visible in the nocturnal nature scene at 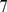 kHz. This is due to a presence of a spatially fixed source generating a sound at this frequency (see figure 2). The most dynamic scene - city center - reveals, as expected, lowest correlations across most of the spectrum.

Interaural level differences ILD were computed separately in each frequency channel. Figure 3 B displays an example ILD distribution (black line) together with a best fitting Gaussian (blue dotted line) and logistic distribution (red dashed line). Logistic distributions provided the best fit to ILD distributions across all frequencies and recorded scenes, as confirmed by the KS-test (see figure S1). ILD distribution at frequency 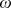 was therefore defined as
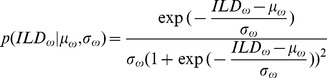
(1)where 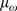 and 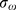 are frequency specific mean and scale parameters of the logistic pdf respectively. Variance of the logistic distribution is fully determined by the scale parameter.

**Figure 4:**
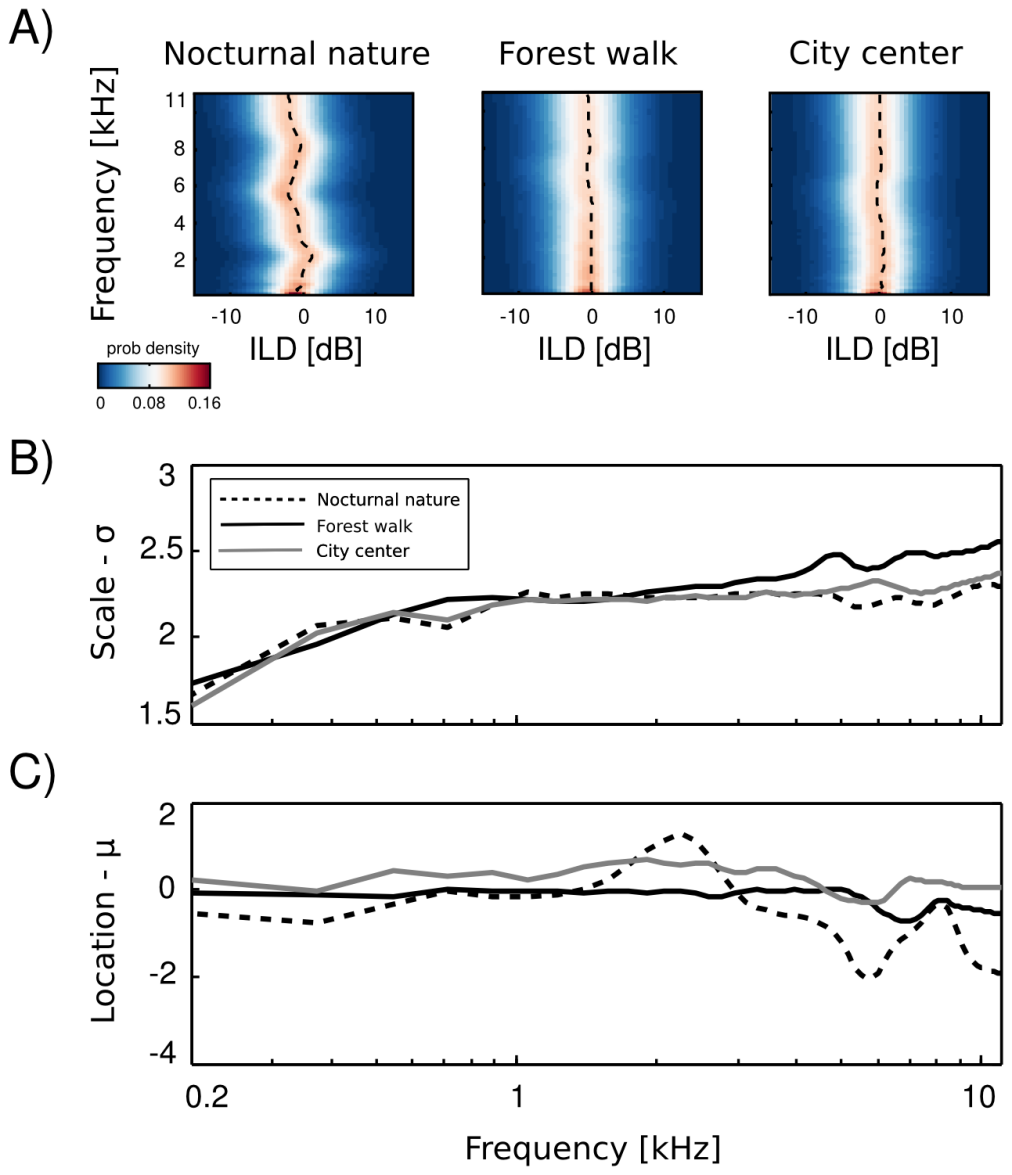
Interaural level difference distributions. A) Histograms plotted as a function of frequency - a strong homogeneity of distributions is visible across recorded scenes and frequency channels. B) The scale parameter 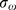 of fitted logistic distributions plotted as a function of frequency C) The location parameter 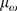 plotted as a function of frequency.

Empirical ILD distributions are plotted on figure 4 A. As can be immediately observed, they preserve similar shape in all frequency channels and auditory scenes, regardless of their type. Scale (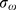) and mean (or location - 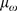) parameters of fitted distributions are plotted as a function of frequency on figures 4 B and C respectively. The mean of all distributions is very close to 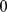 dB in most cases. In two non-static scenes i.e. forest walk and city center deviations from 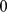 are very small. Marginal ILD distributions of the spatially non-changing scene - nocturnal nature - were slightly shifted away from zero for frequencies generated by a sound source of a fixed position. The difference, however was weak. The scale parameter behaved differently than the mean. In all auditory scenes it grew monotonically with the increasing frequency. The increase was quite rapid for frequencies below 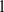 kHz - from 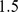 to 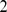. For higher frequencies the change was much smaller and in the 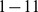 kHz interval 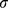 did not exceed the value of 2.5. What may be a surprising observation is the relatively small change in ILD distribution, when comparing high and low frequencies. It is known that level differences become much more pronounced in high frequency channels [19], and one could expect a strong difference with a frequency increase. At least partial explanation can be made, when one observes a close relationship between Fourier spectra of binaural sounds and means of ILD distributions. In a typical, natural setting sound sources on the left side of the head are qualitatively (spectrally) similar to those on the other side, therefore spectral power in the same frequency bands remains similar in both ears. Average ILDs deviate from 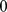 if a sound source was present at a fixed position during the averaged time period. Increase in the ILD variance (defined by the scale parameter 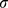) with increasing frequency, can be explained by the filtering properties of the head. While for lower frequencies a range of possible ILDs is low, since large spatial displacements generate weak ILD changes, in higher frequency regimes ILDs become more sensitive to the sound source position hence their variability grows. On the other hand, objects on both sides of the head reveal similar motion patterns and, in this way, reduce the ILD variability, which may account for the small rate of change.

Observed ILD distributions revealed very small variation across different frequencies. The variability was much weaker than what can be predicted from known head filtering properties. Additionally, ILD distributions were quite homogenous across different auditory scenes. This means that neuronal codes for ILDs can optimally represent this cue in very different acoustic environments without necessity of a strong adaptation.

### Interaural phase difference statistics

**Figure 5:**
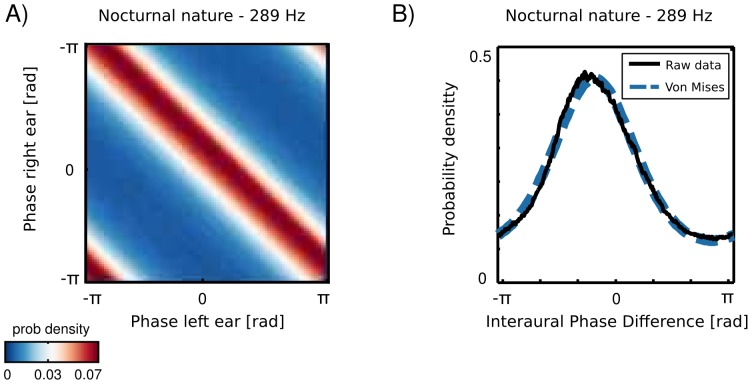
Binaural phase statistics . A) An exemplary joint probability distribution of monaural phases at 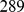 Hz. Data were taken from the nocturnal nature scene. B) An empirical IPD distribution of the same data (black line) plotted with a fitted von-Mises distribution (blue dashed line).



Marginal distribution of a univariate, monaural phase variable over a long time period is uniform, since it periodically assumes all values on a unit circle. An interesting structure appears in a joint distribution of monaural phases (an example is plotted on figure 5 A. Monaural phases reveal dependence in their difference i.e. their joint probability is determined by the probability of the IPD [20]:

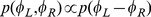
(2)where 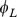 and 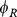 are instantenous phase values in the left and the right ear respectively.

To obtain a parametric description, IPD histograms were fitted with the von Mises distribution as visible in figure 5 B (additional structure was present in IPDs from the forest walk scene - see the following subsection). A distribution of an interaural phase difference in the frequency channel 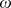 (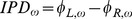), was then given by:
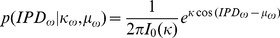
(3)where 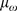 and 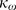 are frequency specific mean and concentration parameters and 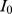 is the modified Bessel function of order 0. In such case, the concentration parameter 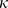 controls mutual dependence of monaural phases [21]. For large 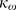 values 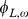 and 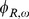 are strongly dependent and the dependence vanishes for 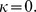


**Figure 6:**
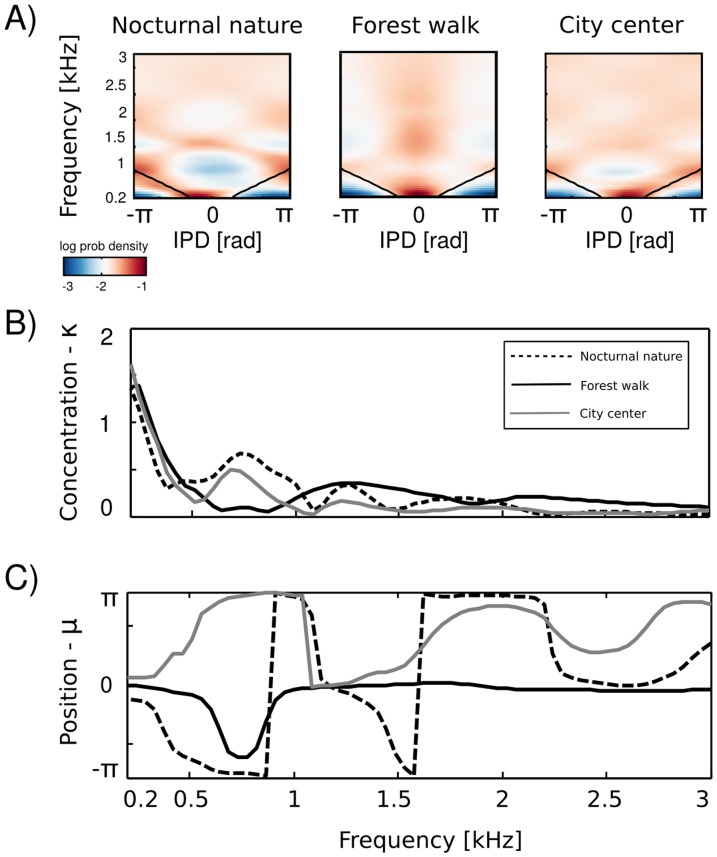
IPD distributions. A) Log-histograms plotted as a function of frequency. Black lines mark the “maximal” IPD limit. B) The concentration parameter 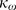 of fitted von-Mises distributions plotted as a function of frequency C) The position parameter 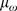 plotted as a function of frequency.

Figure 6 A depicts IPD histograms in all scenes depending on the frequency channel. Thick black lines mark 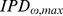 - the “maximal IPD” value i.e. the phase shift corresponding to a time interval required for a sound to travel the entire interaural distance equal to the head diameter (for details see the Materials and Methods section). At low frequencies (below 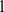 kHz), histograms had a triangular shape. This is a common tendency in IPD distributions, visible across all auditory scenes. Additionally, due to phase wrapping, for frequencies where 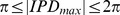 the probability mass is shifted away from the center of the unit circle towards the 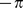 and 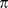 values, which is visible as blue, circular regions. This trend is not present in the forest walk scene, where a clear peak at 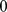 radians is visible for almost all frequencies. Two panels below i.e. figures 6 B and C display plots of 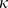 and 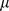 parameters of von Mises distributions as a function of frequency. The concentration parameter 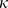 decreased in all three scenes from a value close to 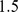 (strong concentration) to below 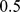 in the interval between 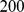 Hz and 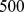 Hz. This seemed to be a robust property in all environments. Afterwards, small 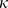 rebounds were visible. For auditory scenes recorded by a static subject i.e. nocturnal nature and city center rebounds occure at frequencies, where 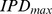 corresponds to 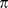 multiplicities (this was again an effect of phase wrapping). The 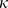 value was higher for a more static scene - nocturnal nature - reflecting a lower IPD variance. For frequencies above 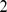 kHz, concentration converged to 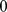 in all three scenes. This means that IPD distributions become uniform and monaural phases mutually independent. The frequency dependence of the position parameter 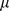 is visible on figure 6 C. Again, division may be made between statically and dynamically recorded scenes. For the latter one, IPD distributions were centered at the 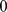 value with an exception at 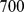 Hz. For two former ones, distribution peaks were roughly aligned along the 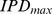 as long as it did not exceed 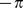 or 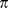 value. One has to note, that for distributions close to uniform (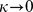), position of the peak becomes an ill defined and arbitrary parameter. We plotted it here, as returned by the estimation algorithm.

**Figure 7:**
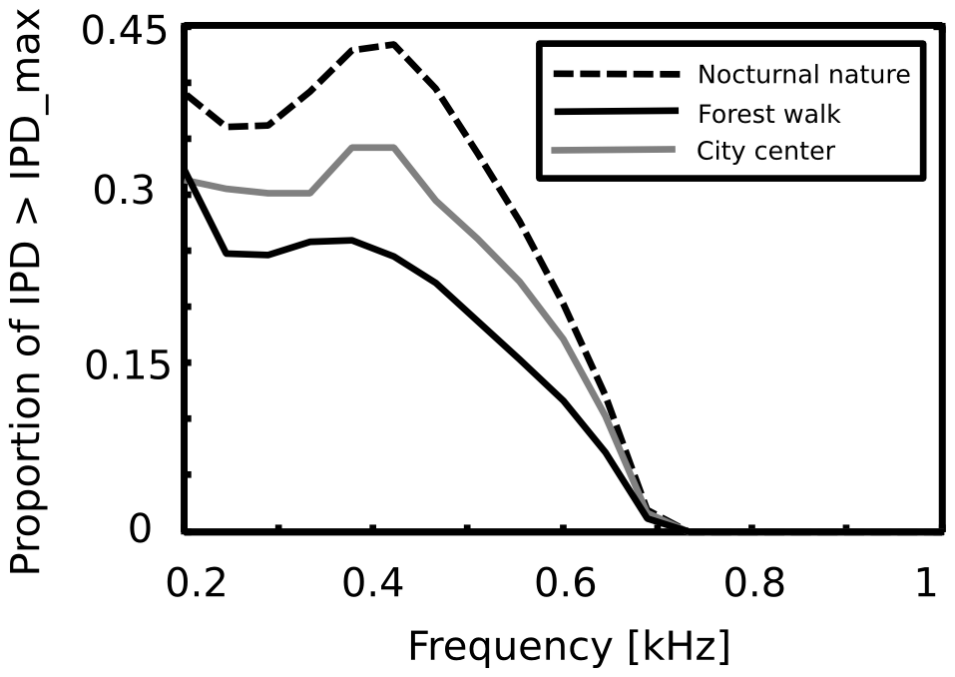
Proportion of IPDs exceeding the “maximal IPD” threshold plotted as a function of frequency. In each auditory environment a substantial amount (up to 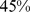 in the 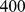 Hz channel of the nocturnal scene) of low-frequency IPDs exceeded the limit imposed by the size of the head. While such IPDs can carry relevant information, they can not be used to identify sound source position without additional transformations.

Thick black lines on figure 6 A mark the “maximal” IPD value (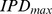), constrained by the size of the organism's head. A single, point sound source in an anechoic environment would never generate an IPD exceeding 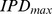. In natural hearing conditions however, such IPDs occur due to presence of two (or more) sound sources at both sides of the head or due to acoustic reflections [15]. The presence of IPDs exceeding the 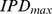 limit is visible on figure 6 as a probability mass lying outside of the black lines. Figure 7 displays a proportion of IPDs larger than the one defined by the head size plotted against frequency. Lines corresponding to three recorded auditory environments lay in parallel to each other, displaying almost the same trend up to a vertical shift. The highest proportion of IPDs exceeding the “maximal” value was present in the nocturnal nature scene. This was most probably caused by a largest number of very similar sound sources (grasshoppers) at each side of the head. They generated non-synchronized and strongly overlapping waveforms. Phase information in each ear resulted therefore from acoustic summation of multiple sources, hence instantenous IPD was not directly related to a single source position and often exceeded the 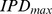 value. Surprisingly, IPDs in the most dynamic scene - city center - did not exceed the 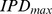 limit as often. This may be due to a smaller number of sound sources present and may indicate that the proportion of “forbidden” IPDs is a signature of a numerosity of sound sources present in the scene. For nocturnal nature and city center scenes the proportion peaked at 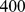 Hz achieving values of 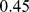 and 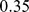 respectively. For a forest walk scene, the peak at 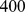 Hz did not exceed the value of 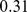 at 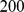 Hz. All proportion curves converged to 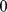 at 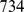 Hz frequency, where 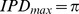.

The percentage of IPDs larger than the value constrained by the head size is another property of auditory scenes, which can not be predicted from head filtering properties or from physics of sound. Our data suggest that since this proportion can be large (up to 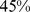), many naturally encountered IPDs do not correspond to single sound sources. This in turn implies that they can not be utilized to identify the sound position in the simplest way suggested by the duplex theory.

#### IPDs of self-generated sounds

**Figure 8:**
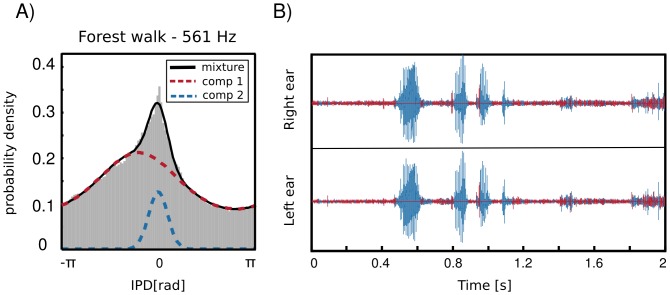
IPD distributions in an auditory scene including self-generated speech. A) An exemplary IPD distribution in the forest walk scene. In addition to a broad “background” component a peak centered at 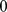 radians is visible. Dashed lines mark components of a fitted von-Mises mixture distribution. B) Results of a sample classification using the fitted mixture model. Intervals were assigned by the algorithm to mixture components of the same color plotted on panel A. Blue intervals include utterances generated by the recording subject.

As already mentioned before, IPD distributions at most of frequency channels in the forest walk scene revealed an additional property, namely a clear, sharp peak at 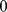 radians. This feature was not present in two other scenes. As an example, IPD distribution at 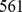 Hz is depicted on figure 8 A. The histogram has a sharp peak close to 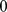, which implies presence of many equal monaural phase values. Zero IPDs can be generated either by sources located at the midline (directly in front or directly in the back) or self-produced sounds such as speech, breathing or loud footsteps.

As visible in figure 8 two components contributed to the structure of the marginal IPD distribution - the sharp “speech component” and the broad “background”. IPD distributions of the forest walk scene were well suited to be modelled by a mixture model. This means that their pdf could be represented as a linear combination of two von Mises distributions in the following way
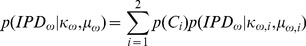
(4)where 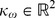 and 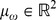 are parameter vectors, 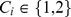 are class labels, 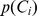 are prior probabilities of a class membership and 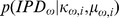 are von Mises distributions defined by equation 3. A fitted mixture of von Mises distributions is also visible in figure 8 A, where dashed lines are mixture components and the continuous black line is the marginal distribution. It is clearly visible that a two-component mixture fits the data much better than a plain von Mises distribution. There is also an additional advantage of fitting such a mixture model, namely it allows to perform classification and assign each IPD sample (and therefore each associated sound sample) to one of two classes defined by mixture components. Since prior over class labels is assumed to be uniform, this procedure is equivalent to finding a maximum-likelihood estimate 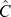 of 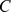




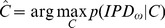
(5)In this way, a separation of self generated sounds from the background can be performed using information from a single frequency channel (if no other sound source is present at the midline). Exemplary results of self-generated speech separation are displayed in figure 8 B. A two-second binaural sound chunk included two self-spoken words with a background consisting of a flowing stream. Each sample was classified basing on an associated IPD value at 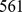 Hz. Samples belonging to the second, sharp component are coloured blue and background ones are red. It can be observed, that the algorithm has succesfuly separated spoken words from the environmental noise.

IPDs are usually considered as cues generated by external sound sources. Our data demonstrate that self-generated sounds such as speech or footsteps, often constitute a dominant component of a natural acoustic scene. They also possess a characteristic statistical signature, which reflects itself in IPD distributions.

### Independent components of binaural waveforms

In previous sections we analyzed statistics of precomputed stimulus features - IPDs and ILDs. In this way we characterized low-order properties of the natural input to binaural circuits in the auditory system. However these results do not allow to draw strong conclusions about mutual dependence of binaural waveforms. This is an important property of the stimulus, since it is informative about the difficulty of the sound localization task in natural environments. If sounds in each ear are highly dependent - it is very likely they are generated by the same source, which can be simply localized using binaural cues. If, however, sound in the left ear is independent from the one in the right ear - this means that each of them is dominated by a different source. In such a case, instantenous cue values can not be directly mapped to a spatial position, and sound localization becomes a complex inference process.

In this section we attempt to estimate the difficulty of sound localization in natural auditory scenes by analyzing mutual dependence of monaural sounds in each scene. To this end we learned high-order patterns present in natural stereo sounds by training Independent Component Analysis (ICA) - a statistical model which optimizes a general-purpose objective - coding efficiency [22].

**Figure 9:**
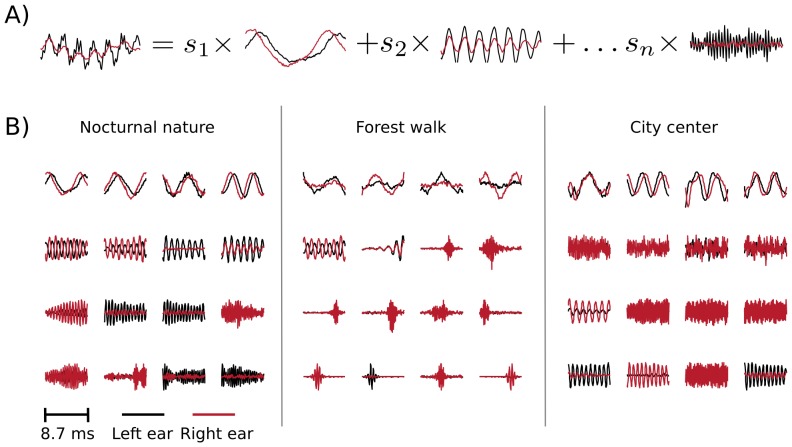
Independent components of natural binaural sounds. A) An explanation of the ICA model. Each epoch of binaural sound (left hand side of the equation) is represented by a linear combination of basis functions (or independent components). Coefficients 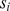 are assumed to be sparse and their joint distribution is equal to the product of marginals. B) Exemplary ICA basis functions from each recorded scene. Nocturnal nature and city center scenes consisted mostly of harmonic sounds and are mostly represented by ICs resembling Fourier bases. The forest walk scene included multiple transient sounds, which gave rise to wavelet-like features.

In the ICA model, short (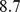 ms) epochs of binaural sounds were represented by a linear superposition of basis functions (or independent components - ICs) multiplied by linear coefficients 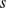 (see figure 9 A. Linear coefficients were assumed to be independent and *sparse* i.e. close to 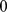 for most of data samples in the training dataset. Basis functions learned by ICA can be interpreted as patterns of correlated inter- and intra-aural variability present in a dataset.

Figure 9 B depicts exemplary basis functions learned from each recording. Each feature consists of two parts, representing signal in the left and in the right ear (black and red colours respectively). Importantly, monaural parts of almost all trained basis functions were well localized in frequency i.e. their Fourier spectra had a prominent peak, in agreement with results presented in [11], [12], [23] (few non-localized features were excluded from the analysis - see Materials and Methods). Features trained on different recordings have characteristic shapes determined by the spectrotemporal composition of auditory scenes. On one hand, the city center scene is modelled by time extended and frequency-localized basis functions (capturing mostly the harmonics of human speech), while on the other the representation of the forest walk scene included temporally localized, instantenous features (induced by transient sounds like wood cracks etc). Spectrotemporal characteristics of learned basis functions (depicted on figure S2) constitute a characteristic property of each auditory scene [11], [23]. Here, we do not analyze them in detail, since this is not the main focus of the current study.

In order to measure how strongly information from each ear contributed to a features encoded by each of the independent components, we computed the peak power ratio (PPR):
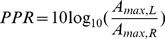
(6)where 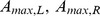 are maximal spectrum values of left and right ear parts of each IC respectively. A large positive PPR value implies a dominance of a left ear sound, while when the PPR is negative the right ear dominates. Values close to 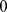 imply a balanced power in each ear. This index is conceptually similar to the binocularity index used to quantify the ocular dominance of real and model visual receptive fields [24], [25].

**Figure 10:**
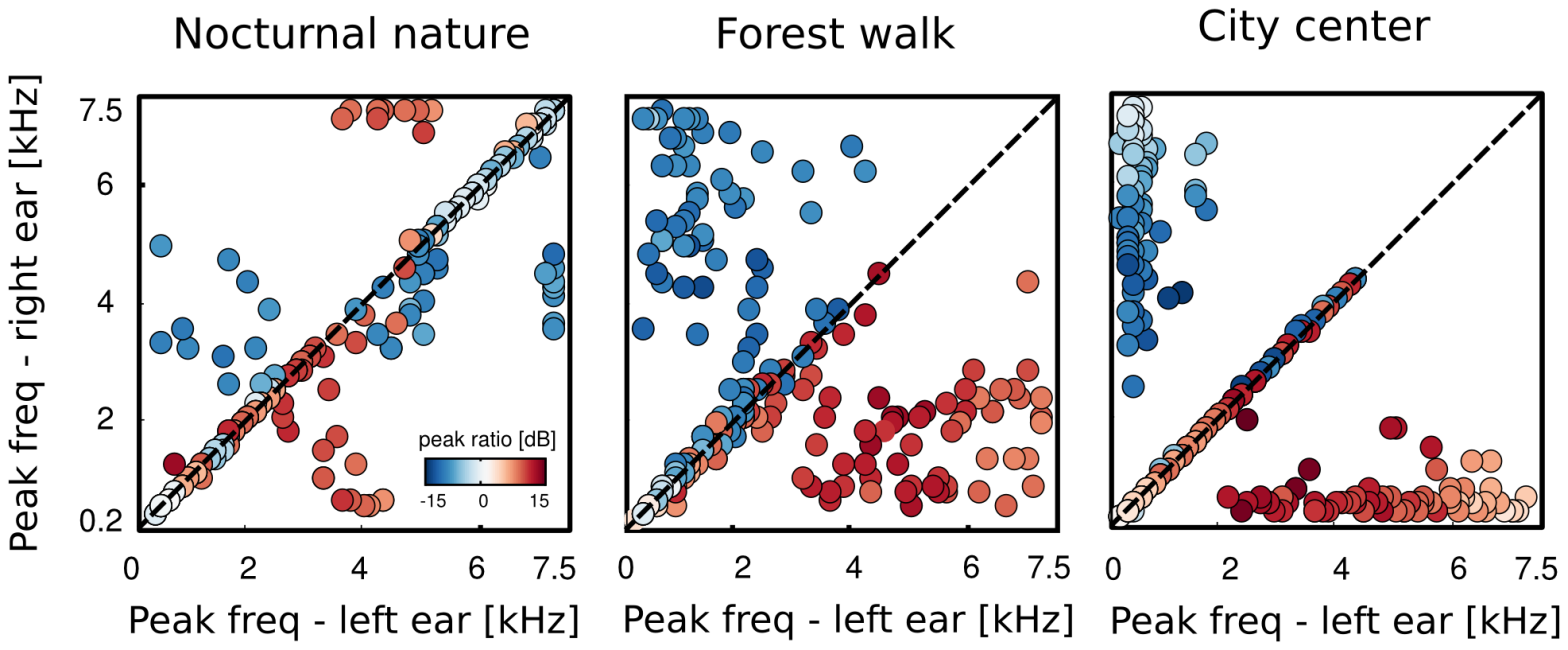
Binaural composition of independent components. Each circle corresponds to a single IC. Horizontal and vertical coordinates are spectral maxima of the left and the right ear parts respectively. Colors encode the peak power ratio. Each pannel depicts one of the recorded scenes.

Figure 10 depicts binaural properties of learned independent components. Each circle represents a single IC. Its vertical and horizontal coordinates are monaural peak frequencies and its color encodes the PPR value. Features which lie along the diagonal can be considered as a representation of “classical” ILDs, since they encoded a feature of the same frequency in each ear and differed only in level. ICs lying away from the diagonal coupled information from different frequency channels in both ears.

Pronounced differences among IC representations of the three auditory scenes are visible on figure 10. Majority (161) of ICs learned from the nocturnal nature scene cluster closely to the diagonal and encode the same frequency in each ear. The basis function set trained on the mostly dynamic scene (city center) separated into three clear subpopulations. Two of them (including 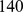 features in total) were monaural. Monaural basis functions were dominated mostly by a single ear, and the contralateral part was of a very low frequency, close to a flat line (a DC component). The binaural subpopulation contained 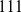 basis functions perfectly aligned with the diagonal. Such separation suggests, that waveforms in both ears were highly independent and modelled by a large separate sets of monaural events. ICA trained on the forest walk scene also yielded a set of basis functions, separable into two populations. Here, the highest number of features - 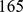 lied off the diagonal and coupled separate frequency channels in each ear. A clear division into two monaural subsets was apparent - almost no IC was characterized by a PPR close to 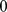.

**Figure 11:**
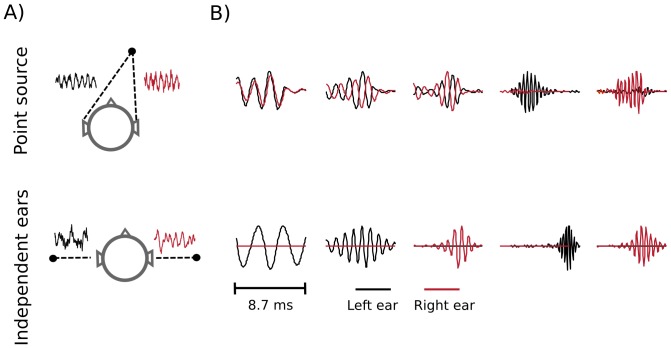
Independent components of simulated data. A) Cartoon illustrations of the generation process of the maximally dependent (top) and the maximally independent (bottom) datasets. B) Exemplary ICs trained on simulated data (point source data - top row, independent ears data - bottom row). If binaural sounds had the same underlying cause, vectors corresponding to each ear captured the signal structure. In the independent ear setting, one of the monaural parts of every IC was always flat.

As data displayed in figure 10 suggest, there is a relationship between interaural redundance and PPR values. In dynamic scenes, where monaural waveforms are generated mostly by independent causes, stereo sounds are best represented by ICs of large absolute PPR values (dominated by a single ear). In order to get a better understanding of this effect, for each recorded scene, we generated two artificial datasets of opposite properties. The first dataset consisted of single, point sources presented in anechoic conditions with zero background noise. It was created by convolving chunks of a recording with human head related transfer functions (HRTFs) from the LISTEN database [26]. This dataset constituted a specific case, where sounds in each ear were maximally dependent given the head filter. In the second dataset the binaural signal was created by drawing two independent sound intervals and treating each of them as an input to a separate ear. The interaural dependence was therefore minimized and emulated a situation in which sounds in each ear originate from separate sources. A cartoon illustration of those two simulations is depicted in figure 11 A. Both - the point source as well as the independent ears dataset were extreme, opposite settings, which do not occur naturally. While in the first one, binaural cues could be directly mapped to a source position, in the second they were spurious and carried no spatial information. Recordings of natural scenes should lay in the space spanned by those two.

ICA was performed on each artificial dataset. Exemplary basis functions learned using sounds from the forest scene are depicted on figure 11 B. Top and bottom rows present ICs trained on point source and independent ears data respectively. Low frequency basis functions representing maximally dependent data (first row) had a very similar value of the spectral peak in each ear, and some of them were shifted in time (encoding an ITD). The power difference increased with frequency growth, due to the head attenuation. ICs encoding independent sounds in each ear, were almost completely monaural i.e. one of the single-ear parts was flat and equal to zero.

**Figure 12:**
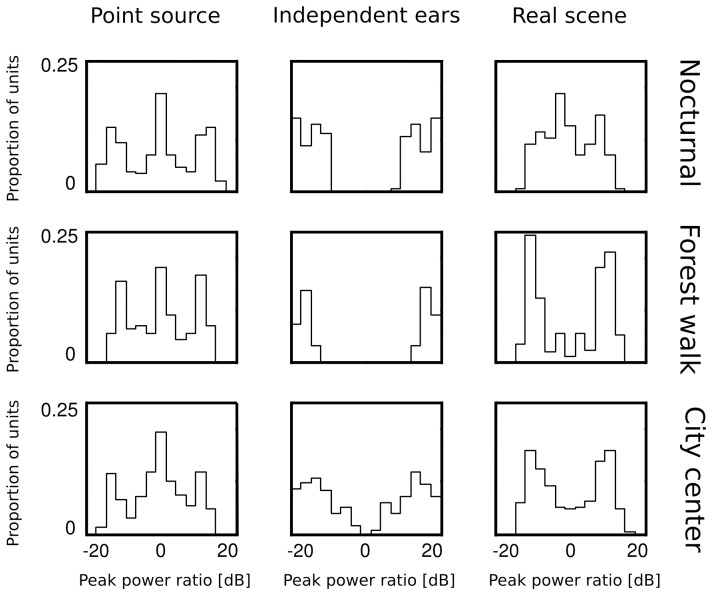
Distributions of peak power ratios of independent components trained on simulated and natural sounds. Columns correspond to datasets (point source, independent ears, natural scene) and rows to recorded environments (nocturnal nature, forest walk, city center). Simulated data gave rise to stereotypical and repetitive PPR distributions. Natural scenes, while being a compromise between simulated environments, were more similar to the independent ear data.

In the next step of the analysis, we computed histograms of the PPR value for each learned IC dictionary. They are depicted in figure 12. A clear, repetitive structure is visible in PPR distributions of ICs trained on artificial datasets. Histograms of point source data (first column) have three peaks - first one at 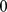 dB and two shorter ones, symmetrically located on either side. The middle peak, located at 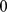 dB corresponds to low-frequency features, which were weakly attenuated by the HRTF, and carried similar power in each ear. High frequency ICs, where sound in one ear was strongly supressed by the head can account for the two symmetric peaks located between 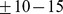 dB. A very different structure is visible in peak ratio histograms of ICs trained on datasets where monaural sounds were independent (middle column). There, two modes were present at extreme PPR values, close to 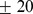 dB. Basis functions learned from those data were dominated by a single ear, while signal in the opposite ear was equivalent to noise fluctuations, giving rise to large absolute PPR values.

Histograms of binaural dominance of natural scene ICs are presented in the third column of figure 12. As expected, they fell in between extremes established by artificial datasets. Both dynamic scenes (recorded in the forest and in the city center) were characterized by PPR distributions highly similar to those obtained from independent ears data. Corresponding histograms consisted of two sharply separated peaks, located away from the 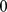 dB point. The distance between the peaks was, however, not as large as for the maximally independent dataset, which implied existence of some binaural dependencies. Importantly, the peak at 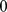 dB visible in maximally dependent datasets was absent in natural scenes. Some binaural features emerged from natural data, however in proportion to monaural ICs their amount was low. This means that monaural sounds were much less redundant than in the simplistic, simulated case. The nocturnal scene, where multiple static sources were recorded by a non-moving subject gave rise to a different PPR distribution. While the 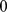 dB maximum was absent as well, the positive and negative peaks were not very sharply separated. Additionally, a clear bias towards the right ear (negative PPRs) was visible. This can be accounted by the fact that this recording was performed in a static environment with a non-moving sound source present close to the right ear. Despite the almost complete lack of motion, even this scene was very different from the simulated point-source one.

The above analysis points to the fact that in a typical auditory environment, sounds in each ear are much stronger dominated by independent acoustic events that can be predicted from considerations of solitary point sources. In such conditions sound localization requires a sophisticated computational strategy and becomes itself a scene-analysis task.

## Discussion

Binaural cues are usually studied in a relationship to the angular position of the generating stimulus [27]–[29]. In probabilistic terms this corresponds to modelling a conditional probability distribution of a cue, given a sound position. Analysis of this relationship in natural environments is a very hard task, since a full knowledge about the spatial configuration of the scene (i.e. position and trajectory of every object) is required in addition to the recorded sound. In the present study we approached binaural hearing from a different perspective - we focused on marginal distributions of naturally encountered binaural sounds.

As a representation of the real sensory world we recorded and analyzed three auditory scenes. Analyzed recordings were very different from each other in terms of spatial configuration as well as sound quality. We have selected them as stereotypes of numerous possible environments consisting of static and moving sources. This diversity increased the likelihood that any other auditory scene typically encountered by a human listener would resemble one of those recorded in the present study. Selected scenes were not free from limitations. Inspection of sound spectra as well as cue statistics revealed slight biases towards the right ear in nocturnal and forest scenes, which may not be the case in all realistic conditions. Moreover, one could invision analyzing a larger amount of recordings performed also in interior, reverberant environments, which are often encountered by humans. Such analysis should allow to draw stronger conclusions about general properties of natural binaural sounds. Despite their differences and limitations, analyzed scenes revealed common features such as the shape of ILD distributions for instance. If all analyzed cases share some statistical property, one may conclude that it should not change strongly in different hearing conditions.

### Binaural cue distributions in natural auditory scenes

Our current understanding of how the nervous system may localize sound sources was primiarily derived from considerations of solitary, point sources of pure frequency sound in noiseless and non-reverberant listening conditions. In such case, knowledge of head filtering properties and analysis based on physics of sound suffices to predict the range of possible binaural cues and their relationship to the position of a generating source.

When considering natural environments, the analytical approach very quickly becomes intractable. In a typical auditory scene, a number of objects unknown to the organism generates interferring sound waves affected by motion and reverberation. Additionally, the number of sources at each side of the head is different. Under such conditions, binaural cues become highly stochastic, and as such should be characterized in statistical terms. In this work we characterized low-order statistics of naturally encountered binaural cues. In many aspects, empirical distributions of natural stimuli deviated from reductionist, analytical predictions. We discuss them below separately for time and level binaural disparities.

#### Interaural level differences

The human head strongly attenuates high frequency tones, acting as a low-pass filter [30]. For this reason, intensity differences between the ears do not carry much information about the position of a low-frequency sound. An ILD becomes informative about the location of a point-source, when the tone frequency exceeds 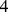 kHz [19]. Based on those observations, one could expect that naturally encountered ILDs are also strongly frequency dependent. This was however, not the case. Empirical ILD distributions were strikingly homogenous across almost entire measured frequency spectrum. Distribution at each frequency was approximately logistic and centered at 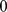 dB. The ILD invariance to a frequency channel is not predictable by the HRTF analysis (although it has been demonstrated before that sound sources proximal to the listener can generate pronounced ILDs also below 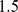 kHz [31], [32]). Weak frequency dependence of natural ILD distributions implies that binaural circuits computing and encoding this cue are exposed to similar patterns of stimulation across large parts of the cochleotopic axis. This allows to make a prediction that similarly tuned neurons encoding both high and low frequency ILDs should be present in the early auditory system. ILD sensitive cells characterized by low best frequencies have been found in the Lateral Superior Olive (LSO) of the cat [33]. Their presence may constitute a manifestation of an adaptation of the binaural auditory system to natural ILD statistics.

A neuron maximizes its coding efficiency (defined by the amount of the stimulus information it conveys), if its tuning curve is equivalent to the cumulative distribution function (CDF) of the naturally encountered stimulus [34]. Since natural ILD distributions are logistic, one can speculate that ILD tuning curves of neurons in the early auditory system should be well approximated by a CDF of this distribution i.e. the logistic function.

In addition to the frequency invariance, ILDs revealed only a small variability across recorded auditory scenes. Despite strong differences between spatial configurations of each scene, ILD distribution parameters fluctuated very weakly. In the nocturnal nature scene, centers of some ILD distributions were slightly shifted away from 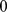 dB, but their shapes were the same. This observation suggests that a very similar tuning curve suffices to efficiently convey the ILD information in various listening conditions. One may conclude that ILD coding neurons do not have to strongly adapt their tuning properties, when an auditory scene changes from one to another. This does not exclude the possibility that adaptation on time scales shorter than analyzed here may still occur. Experimental evidence of a rapid adaptation to fast changes of a cue distribution has been delivered for ILDs [35] (similar effects for ITDs have also been demonstrated in [36]).

#### Interaural phase differences

In anechoic environments, point sources of sound generate interaural time disparities constrained by the head size of the listener - no IPD value should exceed the frequency dependent, physiological threshold. In more complex listening situations larger values can appear, either due to a sound reflection or to a presence of two (or more) desynchronized sound sources [15]. Even though large IPDs can not be directly mapped to a source position, they still may be of high value to the organism. Sound reflections generate reproducible cues and carry information about the spatial properties of the scene [37]. If a large IPD did not arise as a result of a reflection, it means that at least two sound sources contribute to the stimulus at the same frequency. In the latter case, IPDs become a strong source separation cue.

The amount of IPDs larger than the head-imposed threshold is another property of an auditory scene, which can not be derived by the analysis of the head filtering - it has to be estimated from empirical measurements. Present results demonstrate that in low frequency channels large proportions of IPDs exceed the “maximal” value. This was true for to up to 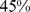 of cues at around 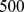 Hz. It means that a large amount of potentially useful signal falls outside of the range predicted by analysis of point sources in echo-free conditions. IPD coding circuits are often exposed to cue values exceeding the threshold when the organism explores the natural environment. In order to retain this information, the auditory system should be adapted to encode IPDs larger than the physiological limit. Interestingly, this notion converges with experimental data. In many mammalian species, tuning curve peaks of IPD sensitive neurons are located outside of the head size constrained range [15]. Moreover, the observed proportion of large IPDs decreased with the frequency increase (since the maximal IPD limit increases with frequency). This observation agrees with the experimental data showing that neurons characterized by the low best frequency are predominantly tuned to IPDs lying outside of the head limit [38]–[41]. Based on the above considerations, we conjecture that tuning to large phase disparities could be also understood as a form of adaptation to the natural distribution of this cue.

The natural auditory stimulus consists not only of external sounds generated by environmental sources, but also of self-generated sounds such as speech. We have found that speech alters the IPD distribution by increasing the number of disparities equal to 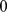 radians. Distribution structure different than in scenes where no self-speech was present implies that binaural stimuli perceived by humans and other vocalizing animals are strongly affected by self generated sounds. This in turn influences activity of cue-coding neurons, since they have to represent IPDs close to 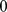 more often. Prior to localizing a source using binaural cues, it has to be determined, whether it is an external source or is it a self-generated one. To a limited extent this can be perfomed using instantenous, single channel IPD values as we have demonstrated here by using a simple mixture model to separate speech from background sounds. The proposed model suggests a possible abstract algorithm, which could be implemented by the nervous system to differentiate between self generated sounds and sounds of the environment. This is a behaviorally relevant task which has to be routinely performed by many animals. One should note that the separation of acoustic sources using binaural cues is a well-known paradigm of computational scene analysis and substantial research has been devoted to it in other contexts (see [42] for an exemplary review).

### Binaural hearing in complex auditory environments

Interaural cues can be directly mapped to a stimulus position only if no other sources of sound overlap with the signal of interest. A natural question to ask is - how often does this happen in the natural environment? This is equivalent to asking - how useful are instantenous, one-dimensional cues to localize typical, real world sources?

Since a direct estimation of a number of auditory objects in real environments is technically very difficult, we approached this problem indirectly. By performing Independent Component Analysis, we learned redundant patterns of natural binaural stimulus. If signals in each ear originated typically from the same source - their dependence was maximized and independent components captured a signal structure in both ears. However, if sounds in each ear were dominated by independent sources, they were best represented by monaural basis functions, where the signal power in one ear was greatly exceeding power in the other one. In order to obtain a frame of reference, we performed the same analysis using simulated datasets. One of them consisted solely of solitary point-sources. Monaural sounds were therefore maximally dependent given the head filter, and sound localization could have been easily performed using simple cues. In the second dataset, sound waves in each ear were completely independent, and binaural cues carried no spatial information.

Basis functions trained on natural auditory scenes had a very different binaural composition than those trained on simulated point sources. In two out of three environments analyzed here, two equinumerous, clearly separated subsets of independent components emerged (in the third one the separation was not so prominent). Each of them was dominated by the signal in only one of the ears. This structure was rather reminiscent of basis functions trained on the artificial, maximally independent data.

These results allow us to conclude that in real-world hearing conditions binaural sound is rarely generated by a single object. Actually, sounds in each ear seem to be dominated by independent environmental causes. In such settings, an inversion of a binaural cue to a sound source position becomes an ill-posed problem. This is because multiple scene configurations can give rise to the same cue value (for instance an ILD equal to 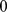 can be generated by a single source located at the midline, or two identical sources symmetricaly located on both sides of the head). A mere extraction of the instantenous cue (as performed by the brainstem nuclei MSO and LSO) is not equivalent to the identification of the sound position. Computation of binaural cues is only a beginning of a complex inference process, whose purpose is to estimate the spatial configuration of an auditory scene [43].

The ICA analysis has yielded a large amount of monaural and a smaller number of binaural features. One can interpret them as model neuronal receptive fields [11]–[13], and ask which role could neurons of such response characteristics play. One possible answer is that while binaural neurons may subserve localization tasks, monaural ones could be used for the purpose of the “better ear listening” i.e. encoding ipsilateral sound sources. On the other hand also monaural sound features similar to those described here can be utilized in further stages of auditory processing to recover spatial information.

## Conclusions

In the present study, we analyzed low-order marginal statistics of binaural cues and estimated the redundance of binaural sounds encountered naturally. In this way, we provided a general statistical characterization of the stimulus processed by the binaural auditory system in natural listening conditions. We have also described our attempt to estimate the complexity of binaural sound localization in the natural environment, based on the Independent Component Analysis. Finally, we have provided stereo recordings of natural scenes, which are availible in the supplementary material, and may be freely used.

In a broad perspective, this study contributes to lines of research attempting to explain functioning of the auditory system by analyzing natural stimuli. Further understanding of binaural hearing mechanisms will require a systematic analysis of high order stimulus statistics. This is a subject of future research.

## Materials and Methods

### Recorded scenes

The main goal of the study was to analyze cue distributions in different auditory environments. To this end, three auditory scenes of different spatial configuration and acoustic properties were recorded. Each of the recordings lasted 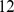 minutes.

1. **Nocturnal nature** - the recording subject sat in a randomly selected position in the garden during summer evening. During the recording the subject was keeping his head still, looking ahead, with his chin parallel to the ground. The dominating background sound were grasshopper calls. Other acoustic events included sounds of a distant storm and a few cars passing by on a near-by road. The spatial configuration of this scene did not change much in time - the scene was almost static.
2. **Forest walk** - this recording was performed by a subject freely moving in the wooded area. The second speaker was present, engaged in a free conversation with the recording subject. In addition to speech, this scene included environmental sounds such as flowing water, cracks of broken sticks, leave crunching, wind etc. Binaural signal was affected not only by the spatial scene configuration, but also by head and body motion patterns of the recording subject.
3. **City center** - the recording subject sat in a touristic area of an old part of town, fixating the head as in the previous case. During the recording many moving and static human speakers were present. Contrasted with the previous example, the spatial configuration of the scene varied continuously.

Two of the analyzed auditory scenes (nocturnal nature and city center) were recorded by a non-moving subject, therefore sound statistics were unaffected by listener's motion patterns and self generated sounds. In the third scene (forest walk) the subject was moving freely and was speaking sparsely. Scene recordings are publicly available at the following URL: http://figshare.com/articles/Statistics_of_Natural_Binaural_Sounds_Supplementary_Material/1157161

### Binaural recordings

Recordings were performed using the Soundman OKM-II binaural microphones which were placed in the left and the right ear channels of the recording subject. Soudmann DR2 recorder was used to simultaneously record sound in both channels in an uncompressed wave format at 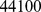 Hz sampling rate. The circumference of the recording subject's head was equal to 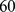 cm.

### Frequency filtering and cue extraction

Prior to analysis, raw recordings were down-sampled to 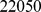 Hz sampling rate. The filtering and cue extraction pipeline is schematically depicted in figure 1.

To obtain a spectral decomposition of the signal, sound waveforms from each ear were transformed using a filterbank of 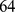 linear gammatone filters. Filter center frequencies were linearly spaced between 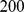 and 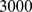 Hz for IPD analysis and 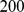 and 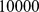 Hz for ILD analysis. Biological cochlear filters are spaced logarithimcally. Here however, a linear spacing was utilized. This resulted in a more uniform coverage of the frequency range than in the case of a biologically plausible filterbank. Within the limits of the analysis performed here, results should not be significantly different when using different filterbanks for preprocessing.

A Hilbert transform of each frequency channel was performed. In result, instantenous phase 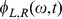 and amplitude 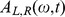 were extracted, separating level and time information. Instantenous binaural cue values were computed in corresponding frequency channels 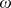 from both ears according to the following equations:
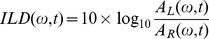
(7)

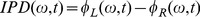
(8)


IPDs with absolute value exceeding 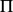 were wrapped to a 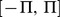 interval. Time series of IPD and ILD cues obtained in this way in each frequency channel were subjected to further analysis.

### Computation of the “maximal” IPD value

In each frequency channel 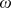, the maximal IPD value constrained by the head size (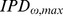) was computed in the following way. The head shape was assumed to be spherical. Given this assumption, the time period required by the sound wave to travel the distance between the ears is equal to:
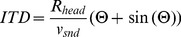
(9)where 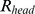 is the head radius, 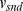 the speed of sound and 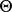 the angular position of the sound source measured in radians from the midline. The ITD is maximized for sounds located directly oposite to one of the ears, deviating from the midline by 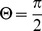. 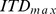 becomes



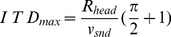
(10)The maximal IPD was computed separately in each frequency channel 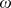


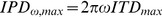
(11)


The above calculations assume a spherical head shape, which is a major simplification. It was, however, satisfactory for the sake of the current analysis.

### Independent Component Analysis

Independent Component Analysis (ICA) is a family of algorithms which attempt to find a linear transformation of the data which minimses redundancy [24]. Given the data matrix 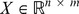 (where 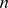 is the number of data dimensions and 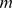 number of samples), ICA finds a filter matrix 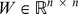, such that:
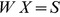
(12)where columns of 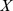 are data vectors 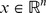, rows of 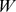 are linear filters 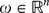 and 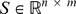 is a matrix of latent coefficients, which according to the assumptions are marginally independent. Equivalently the model can be defined using a basis function matrix 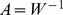, such that: 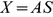
(13)The columns 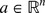 of the matrix 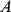 are called basis functions. In modelling of neural systems they are usually interpreted as linear receptive fields forming an efficient code of the training data ensemble [22]. Each data vector can be represented as a linear combination of basis functions 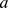, multiplied by linear coefficients 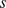 according to the equation 14.
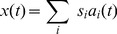
(14)where 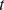 indexes the data dimensions. The set of basis functions 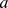 is called a dictionary. ICA attempts to learn a linear, maximally non-redundant code, hence the latent coefficients 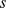 are assumed to be statistically independent i.e.



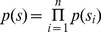
(15)The marginal probability distributions 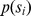 are typically assumed to be sparse (i.e. of high kurtosis), since natural sounds and images have an intrinsically sparse structure [44] and can be represented as a combination of a small number of primitives. In the current work we assumed a logistic distribution of the form:
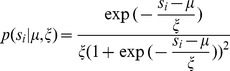
(16)with position 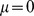 and the scale parameter 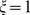. Basis functions were learned by maximizing the log-likelihood of the model via gradient ascent [24].

Prior to ICA learning, the recordings were downsampled to 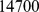 Hz sampling rate. A training dataset was created by randomly drawing 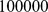 intervals each 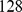 samples long (corresponding to 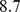 ms). The sampling rate and the length of the time interval were equal to those used in [11].

After learning, we rejected spectrally non-localized independent components as they typically reflect noise, not data structures [12]. All basis functions for which the sum of two spectral maxima in each ear constituted less than 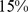 total power were removed. This resulted in 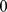, 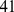 and 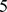 components rejected from the nocturnal, forest and city scenes respectively.

### Generation of artificial data

Two artificial datasets corresponding to extreme cases of binaural redundance were generated using sounds from each recorded scene. Binaural recordings were transformed to a single channel by averaging sound in both ears. Point-source datasets were created by drawing random intervals of the mono recording and convolving them with Head Related Transfer Functions (HRTFs) corresponding to one of the 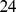 positions (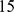 degree spacing) on a circle surrounding the head. Human HRTFs were taken from the publically available LISTEN database [26]. Maximally independent datasets were created by independently sampling two epochs of sound and treating each of them as an input to one of the ears. Each dataset consisted of 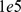 samples of binaural sound, each 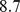 ms long. Recorded and simulated datasets had the same Fourier spectra, but a very different dependence structure.

## Supporting Information

Figure S1
**Results of the goodness of fit test for ILD distributions.** Curves plot p-values of the Kolmogorov-Smirnov test. The purpose of the test was to compare empirical ILD distributions at each frequency with the logistic distribution. The null hypothesis was that both distributions are equal. Black dots mark frequency channels, where one of the p-values was below 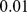. As visible on the plot in all except 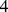 cases (across all scenes and frequencies), the test did not allow for rejection of the null hypothesis at 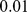 confidence level.(TIFF)

Figure S2
**Spectrotemporal representation of independent components.** Panels in the left and right columns correspond to the left and the right ear IC parts respectively. Rows correspond to auditory scenes. To obtain a time-frequency representation, Wigner-Ville distributions were computed for each monaural part of each IC. This transformation localizes energy of a temporal waveform on a time-frequency plane. Each component is represented by the iso-probability contour of the Wigner-Ville distribution corresponding to 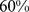 of the total energy. Monaural vectors belonging to the same IC are plotted in the same color.(TIFF)
