# Supplementary figures and images for: Statistics of Natural Binaural Sounds
Source: PLoS One. 2014 Oct 6;9(10):e108968. doi: 10.1371/journal.pone.0108968 (PMC4186785; doi:10.1371/journal.pone.0108968)

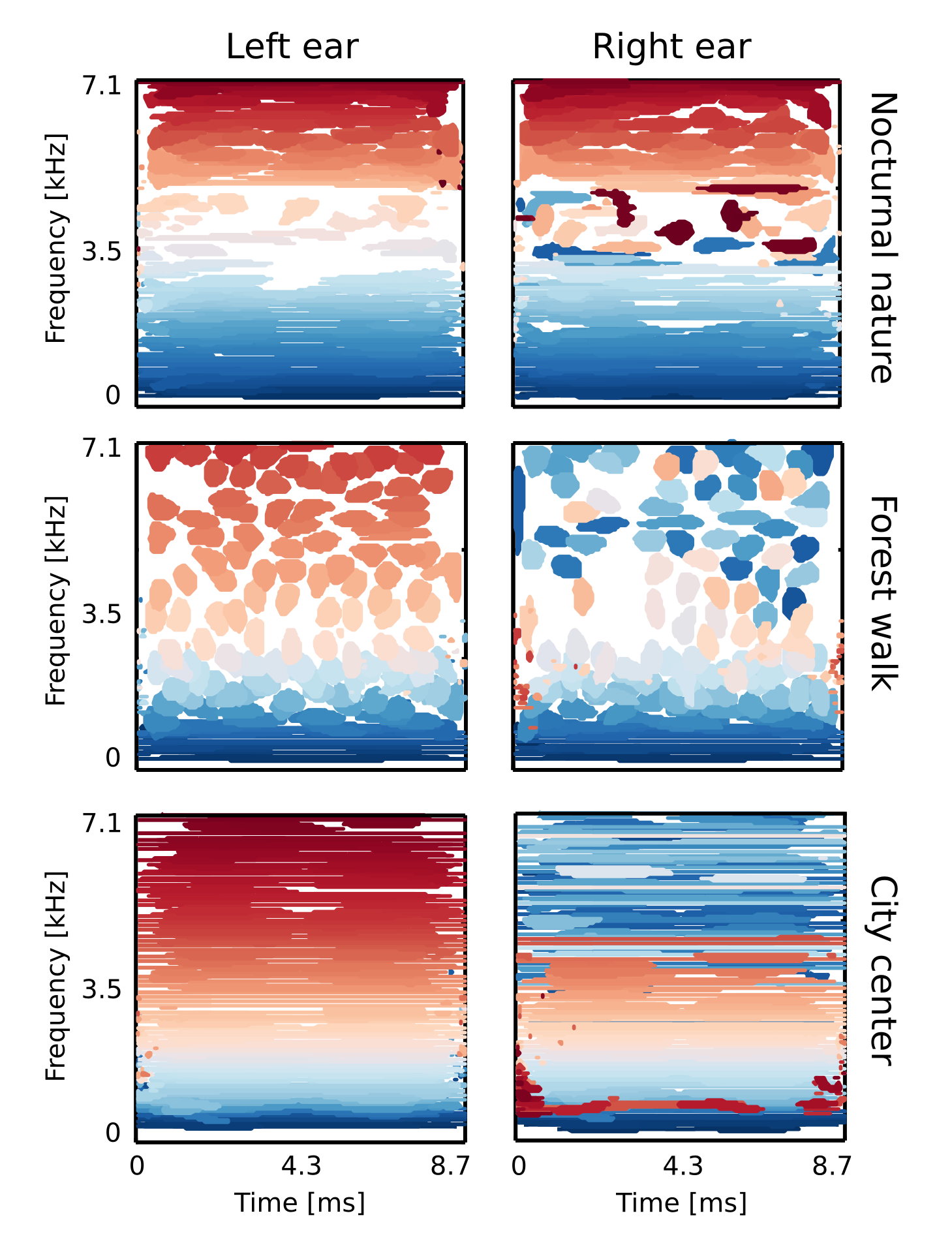

Supplement: Figure S2 — Spectrotemporal representation of independent components. Panels in the left and right columns correspond to the left and the right ear IC parts respectively. Rows correspond to auditory scenes. To obtain a time-frequency representation, Wigner-Ville distributions were computed for each monaural part of each IC. This transformation localizes energy of a temporal waveform on a time-frequency plane. Each component is represented by the iso-probability contour of the Wigner-Ville distribution corresponding to of the total energy. Monaural vectors belonging to the same IC are plotted in the same color. (TIFF) [file pone.0108968.s002.tiff]
